# Melanin Transfer in the Epidermis: The Pursuit of Skin Pigmentation Control Mechanisms

**DOI:** 10.3390/ijms22094466

**Published:** 2021-04-24

**Authors:** Hugo Moreiras, Miguel C. Seabra, Duarte C. Barral

**Affiliations:** iNOVA4Health, CEDOC, NOVA Medical School, NMS, Universidade NOVA de Lisboa, 1169-056 Lisboa, Portugal; hugo.moreiras@ucdconnect.ie (H.M.); miguel.seabra@nms.unl.pt (M.C.S.)

**Keywords:** melanin, melanosome, melanocore, melanocyte, keratinocyte, skin pigmentation, melanokerasome

## Abstract

The mechanisms by which the pigment melanin is transferred from melanocytes and processed within keratinocytes to achieve skin pigmentation remain ill-characterized. Nevertheless, several models have emerged in the past decades to explain the transfer process. Here, we review the proposed models for melanin transfer in the skin epidermis, the available evidence supporting each one, and the recent observations in favor of the exo/phagocytosis and shed vesicles models. In order to reconcile the transfer models, we propose that different mechanisms could co-exist to sustain skin pigmentation under different conditions. We also discuss the limited knowledge about melanin processing within keratinocytes. Finally, we pinpoint new questions that ought to be addressed to solve the long-lasting quest for the understanding of how basal skin pigmentation is controlled. This knowledge will allow the emergence of new strategies to treat pigmentary disorders that cause a significant socio-economic burden to patients and healthcare systems worldwide and could also have relevant cosmetic applications.

## 1. Introduction

The skin is the largest organ of the human body and fulfills essential functions in protection against external aggressions, including ultraviolet radiation (UVR) and infection, while maintaining water and body temperature homeostasis [1]. The pigmentary system is an essential part of these functions and is responsible for the variation in skin color traits within and between populations. Indeed, skin pigmentation is one of the most distinct and noticeable individual characteristics. We now understand that the correlation with protection from UVR is the basis of the evolution of skin pigmentation variation in humans. Two main types of cells compose the skin epidermis: the keratinocytes, the most abundant cells, which are present in all the layers of the epidermis and produce keratin to protect epithelial cells from mechanical and non-mechanical stress; and melanocytes, which are present at the basal layer of the epidermis and produce the protective pigment melanin [1,2]. Melanocytes are neural crest-derived cells arising from the dermal melanoblast lineage that migrate to the epidermis during embryonic development [3,4,5]. Keratinocytes continuously proliferate, migrate, and differentiate toward the upper layers of the epidermis, forming five layers or *strata*: *stratum basale*, *stratum spinosum*, *stratum granulosum*, *stratum lucidum* and *stratum corneum* [6,7]. These five layers are only present in thick skin, while in thin skin, which covers most of the human body, the *stratum lucidum* is not observed. One melanocyte can contact up to 40 viable keratinocytes through its dendrites, forming the so-called epidermal-melanin unit [8,9].

Skin pigmentation results from three different processes: (i) melanin biogenesis and transport within melanocytes; (ii) melanin transfer from melanocytes to keratinocytes; and (iii) melanin internalization and processing by keratinocytes. Within keratinocytes, melanin accumulates in the supranuclear region, protecting the nuclear genetic material from UVR-induced damage [10].

Different classifications are used to categorize humans according to their skin pigmentation. These include the racial groups Caucasoid, Negroid, and Mongoloid and the Fitzpatrick scale of six phototypes (I to VI), based on skin color and the visible response to UVR stimulation [10,11]. Since these criteria do not represent the diversity of skin colors in humans, a new method of classification is being implemented using the Individual Typology Angle (ITA). This classification method is based on colorimetric parameters that better represent skin color diversity in each person [10]. Notably, racial diversity cannot be attributed to a higher number of melanocytes in darker skins [10]. Instead, differences in skin color are thought to be due to the type (eumelanin vs. pheomelanin), distribution and amount of melanins, as well as the size, number, and type of melanin-containing compartments within keratinocytes. Melanins are a group of biopolymers that are synthesized from tyrosine in melanocytes, within lysosome-related organelles (LROs) called melanosomes. Dark eumelanins (ranging from brown to black pigment) are the product of successive hydroxylation, oxidation, and carboxylation reactions, whereas the formation of pheomelanins (ranging from yellow to red pigment) requires at least one cysteine-dependent reduction step [12,13]. The fate of melanin once taken up by keratinocytes is much less studied, including the compartment in which it resides. We propose to call this compartment melanokerasome, as it contains melanin and is formed within keratinocytes.

In dark skins, melanokerasomes have larger pigment cores and are individually distributed throughout the cytoplasm of keratinocytes, whereas in light skins they have smaller cores and are aggregated in clusters [14]. The ratio of clusters to single melanin granules decreases as skin phototype increases [15,16,17]. Of note, lightly pigmented skins do not present melanin in the upper layers, while darkly pigmented skin maintains the pigment in those layers [14]. These observations suggest that one critical factor, among others, in the determination of different skin phototypes is the melanin transfer mechanism between pigment-producing melanocytes and pigment-recipient keratinocytes.

## 2. Melanin Transfer between Melanocytes and Keratinocytes

Melanosomes are considered LROs because they share proteins with lysosomes, are acidic in early stages, and are secreted from melanocytes [18,19,20]. Melanin synthesis and melanosome transport within melanocytes are well characterized. Melanosome biogenesis is divided in four organelle stages [18,20]. During stage I, there is formation of non-pigmented pre-melanosomes containing internal membranous vesicles that resemble early/sorting endosomes [21]. This compartment is characterized by intraluminal proteinaceous fibrils that begin to form at this stage and are completed by stage II [22]. Melanin synthesis and melanosome maturation begin with the acquisition of an elliptical shape at the end of stage II. Stage III is characterized by melanin deposition on the amyloid fibrils, resulting in their thickening and darkening, until it becomes fully melanized achieving the stage IV, when it is considered a fully mature melanosome [19,20,23].

To be transferred to keratinocytes, melanosomes need to be transported from the perinuclear region to melanocyte dendrites. This is thought to occur in a two-step cooperative process, whereby melanosomes first employ a long-range bidirectional transport dependent on microtubules. Indeed, melanosomes move in a kinesin 2-dependent manner to the cell periphery, where they become tethered to the actin cytoskeleton [24,25]. At the periphery, melanosomes exhibit a short-range movement on the cortical actin network that is dependent on the tripartite complex formed by Myosin Va, Melanophilin, and Rab27a [26,27]. Melanosome distribution within melanocytes is postulated to result from a competition between microtubule- and actin-dependent transport [28]. A recent report suggests that inter-melanosome repulsion based on actin filament dynamics could maintain the spreading of melanosomes throughout the cytoplasm, without the need for microtubule-dependent transport [29]. Furthermore, another study showed that in retinal pigment epithelium (RPE) cells, microtubule transport is essential for fast long-range transport of melanosomes and that cytoplasmic dynein is indispensable for the passage of these organelles to an actin filament-dependent transport [30]. Therefore, the debate on this topic is still ongoing, as evidenced by the suggestion for a role of dynein components in melanosome maturation, position, and transfer to neighboring keratinocytes [31].

**Figure 1:**
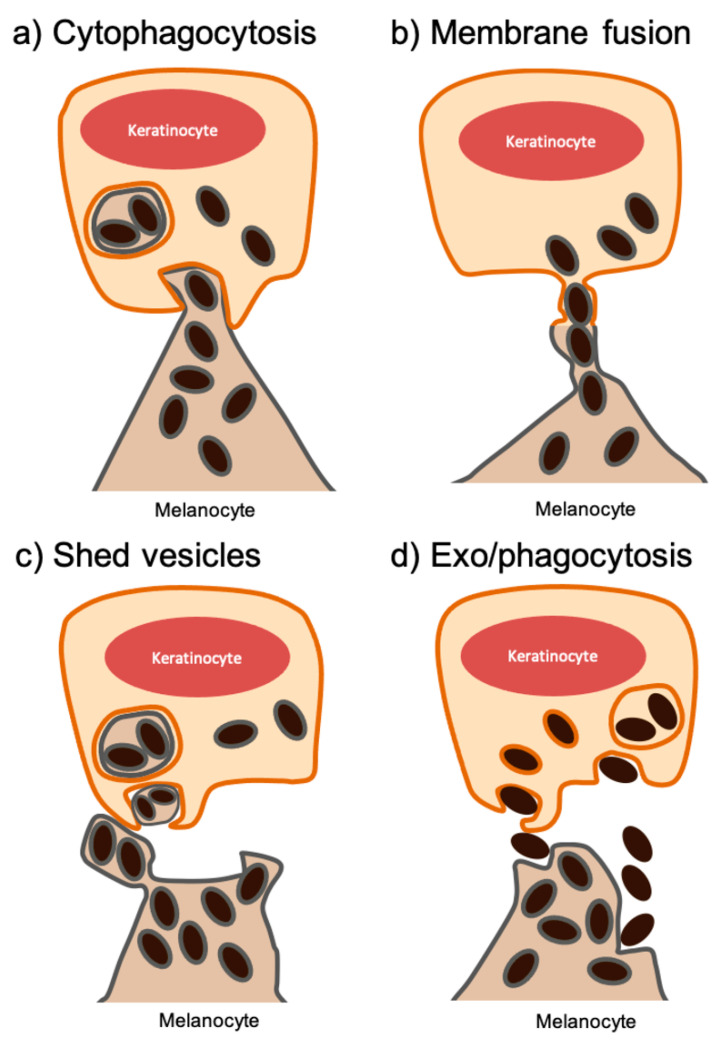
Proposed models for melanin transfer. (**a**) Cytophagocytosis: a melanocyte dendrite is phagocytosed, forming a phagolysosome from which melanin granules disperse through the cytoplasm of keratinocytes. (**b**) Direct membrane fusion: the plasma membranes of both cells fuse, creating a nanotube that allows the passage of melanosomes. (**c**) Shed vesicles: melanosomes are shed in vesicles from the melanocyte, which fuse with the keratinocyte plasma membrane or are phagocytosed. (**d**) Coupled exo/phagocytosis: melanin is secreted to the intercellular space through the fusion of the melanosome membrane with the melanocyte plasma membrane and is then phagocytosed by the keratinocyte.

The molecular mechanisms controlling melanin transfer from melanocytes to keratinocytes remain controversial as there is yet no consensus in the field. There are currently four proposed models to explain this process: (a) cytophagocytosis of melanocyte dendrite tips by keratinocytes; (b) direct membrane fusion between melanocytes and keratinocytes, establishing filopodia through which melanosomes are transferred; (c) transfer of shed melanosome-loaded vesicles from melanocytes, followed by internalization by keratinocytes; and (d) exocytosis of the melanin core by melanocytes and subsequent internalization by keratinocytes (Figure 1). We will examine each of the models in more detail below.

### 2.1. Cytophagocytosis of Melanocyte Dendrite Tips by Basal Keratinocytes

This model is based on the phagocytosis of a portion of a melanocyte by a keratinocyte and was first proposed after the observation by electron microscopy (EM) of melanocyte dendrite tips within keratinocytes cultured in vitro [32]. Generally, this model could be divided in four steps. In the first step, the melanocyte extends its dendrites, contacting a surrounding keratinocyte. The keratinocyte then engulfs the melanocyte dendrite tip through ruffling cytoplasmic projections. Although phagocytosis is usually associated with specialized phagocytic cells, like macrophages, neutrophils, and monocytes, keratinocytes also possess phagocytic ability, which was shown both *in vitro* and *in vivo* [33,34]. In the second step, the melanocyte dendrite tip is pinched off, resulting in the formation of a cytoplasmic vesicle filled with melanosomes. Therefore, this model postulates that melanin becomes surrounded by three membranes: the melanosome membrane and those derived from the melanocyte and keratinocyte plasma membranes [35,36]. During the third step, newly formed melanokerasomes fuse with lysosomes, forming a phagolysosome, leading to the degradation of all three internal membranes. Finally, the melanized phagolysosome fragments into smaller vesicles containing aggregates or single melanin granules dispersed in the cytoplasm [32,37]. In most studies, however, melanin is found surrounded by a single membrane within keratinocytes. Therefore, future studies should uncover evidence that would demonstrate this fast degradation of the melanocyte-derived membranes, as proponents of this model suggest that it occurs faster than the limiting membrane of the melanokerasome [35].

### 2.2. Membrane Fusion of Melanocyte and Keratinocyte Membranes

This model of transfer proposes that the melanocyte and keratinocyte plasma membranes fuse, forming filopodia that connect the cytoplasm of both cells and allow melanosome transfer [38]. In this model, melanosomes retain their original membrane, being transferred to keratinocytes as single membrane-bound organelles. Although melanocyte filopodia have been observed to attach to the surface of keratinocytes [39,40] and transmission EM studies also support this model [41], convincing evidence of melanosome transfer through these structures is still lacking. Interestingly, Rab17 was shown to be required for melanocyte filopodia formation and its depletion leads to melanin accumulation at the cell periphery [40]. Moreover, Myosin X and *N*-methyl-D-aspartate (NMDA) receptor inhibition were observed to inhibit filopodia formation and impair melanin transfer to keratinocytes [39,42]. In this model, E-cadherin seems to be crucial for melanocyte filopodia formation after UVR stimulation and, consequently, melanin transfer. Indeed, the Tobin group showed that an increase in extracellular calcium levels induces a dose-dependent filopodia formation and pigment transfer, through an increase in β-catenin, CDC42, Myosin X, and E-cadherin [43]. Nevertheless, it remains a possibility that this model and the one based on cytophagocytosis are variations of the same mechanism. Furthermore, studies from the Raposo and our laboratories failed to identify melanosome membrane markers within keratinocytes by immunofluorescence and EM [14,44].

### 2.3. Transfer of Shed Melanosome-Loaded Vesicles

This model postulates that melanosome-loaded vesicles are released from melanocytes to the extracellular space, being subsequently phagocytosed by keratinocytes. This phenomenon was first observed in human melanoma cells [45] and later in *Xenopus laevis* [46], originating a new model for melanin transfer. Similar to the cytophagocytosis model, the melanokerasomes are predicted to be composed of three membranes: the melanosome membrane and the plasma membranes of the melanocyte and the keratinocyte. The proposed mechanism comprises four sequential steps: (1) packaging of multiple melanosomes in a single vesicle; (2) shedding of these vesicles enclosed by the melanocyte plasma membrane; (3) internalization by keratinocytes of these vesicles through phagocytosis; and (4) release of the individual melanosomes into the cytoplasm of keratinocytes. More recently, *in vivo* evidence of the occurrence of melanosome shedding in chicken embryonic skin samples was provided, implicating the Rho small GTPase family in melanocyte membrane remodeling before vesicle release [47]. Moreover, flow cytometry analysis of human melanoma cells showed a population of vesicles thought to be the shed melanosome-loaded vesicles [48]. Indeed, vesicles loaded with multiple melanosomes were found in the culture medium of melanoma cells, which is inconsistent with the cytophagocytosis and membrane fusion models [49]. Additionally, EM studies revealed that keratinocytes incorporate aggregates of melanosomes enclosed by a double membrane [45,47,49,50]. Furthermore, protease-activated receptor (PAR)-2 stimulation was reported to increase the transfer of these vesicles [51,52,53]. Importantly, PAR-2 has been shown to mediate melanin uptake in human keratinocytes *in vivo* and *in vitro*, since its activation stimulates melanin transfer through increased phagocytosis in keratinocytes [34,54]. Finally, upon internalization, gradual degradation of the membranes within melanokerasomes occurs [47].

### 2.4. Coupled Exocytosis of the Melanin Core by Melanocytes and Phagocytosis by Keratinocytes

This model proposes that melanin transfer is accomplished by fusion of the melanosome membrane with the melanocyte plasma membrane. This results in the release of the melanosome core, termed melanocore, followed by phagocytosis by neighboring keratinocytes. Importantly, this gives rise to melanokerasomes surrounded by a single membrane derived from the keratinocyte plasma membrane. This model was first proposed in 1964 after the observation of extracellular melanin in human skin and hair, which was proposed to be later internalized by neighboring keratinocytes as individual granules or as melanin clusters [55]. Additionally, naked melanin was detected in the media of co-cultures of human melanocytes and keratinocytes [56]. Moreover, EM studies of human skin found melanocores devoid of any membrane in the extracellular space before being phagocytosed, individually or in groups, by keratinocytes [14,44]. Furthermore, we showed that melanin is surrounded by a single membrane upon internalization by keratinocytes and this membrane is mostly devoid of the melanosome membrane protein tyrosinase-related protein (TYRP)-1 [44]. Numerous factors were demonstrated to enhance melanin transfer between melanocytes and keratinocytes. Among them, α-Melanocyte stimulating hormone (MSH) and endothelin-1 (ET-1) show the ability to induce melanosome secretion from melanocytes [33,56,57]. Our studies found evidence that Rab11b depletion in melanocyte-keratinocytes co-cultures leads to an impairment in melanin transfer by blocking melanin secretion from melanocytes [44,58]. Thus, these findings further support the model of coupled exo/phagocytosis. We note that EM studies of skins showing melanokerasomes containing membrane-bound melanosomes have never been reported in mammals to the best of our knowledge. Furthermore, we recently showed that melanocore uptake is dependent on the presence and activation of PAR-2, in contrast with melanosomes [54,59]. Therefore, we hypothesize that the way melanin is presented to keratinocytes influences how it is further processed and suggest that, in mammals, PAR-2-dependent phagocytosis is the predominant mechanism.

Despite many studies that attempted to dissect the mechanism of intercellular melanin transfer in the skin, the controversy in the field remains. Recently, the Raposo group showed a critical role for caveolae in melanin transfer from melanocytes to keratinocytes both in co-cultures and reconstructed human epidermises [60]. Although this observation does not support any melanin transfer model *per se*, it shows the importance of melanocyte signaling and membrane remodeling in skin pigmentation. Therefore, considering the existing evidence in the literature, it cannot be excluded that multiple mechanisms of melanin transfer co-exist to achieve skin pigmentation.

## 3. Melanin Secretion from Melanocytes

**Figure 2:**
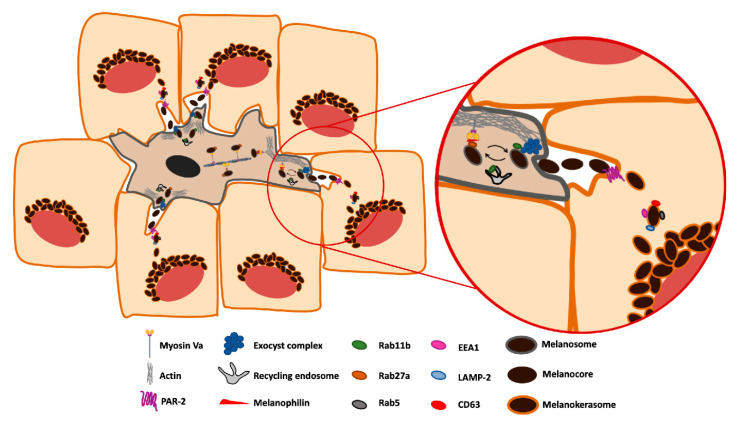
Schematic representation of melanin transfer between melanocytes and keratinocytes in the skin through exo/phagocytosis. After melanosome maturation and transport to the periphery of the melanocyte, Rab11b is recruited to melanosome membranes and the organelle interacts with the exocyst complex to allow melanocore secretion to the extracellular space. Following secretion, melanocores are phagocytosed by keratinocytes in a PAR-2 dependent manner. After internalization by keratinocytes, melanocores follow the endocytic pathway and colocalize with early and late endosomal markers, being stored in mildly acidic and degradative compartments, which we named melanokerasomes, that allow melanin to resist degradation.

Previous reports by us and others presented evidence supporting the exocytosis of melanocores, consistent with the model of exo/phagocytosis [14,44,54,58]. Indeed, EM analysis of human skin samples revealed the presence of naked melanin in the extracellular space between melanocytes and keratinocytes. We also found evidence that the final steps of melanocore exocytosis from melanocytes are mediated by the small GTPase Rab11b, before the transfer to keratinocytes [44]. Interestingly, Rab11 has been shown to play a role in the exocytosis of cytotoxic T-lymphocyte lytic granules, which are also LROs [61]. A critical step in exocytosis is the tethering of vesicles to the plasma membrane, which requires tethering factors such as the exocyst. The exocyst is an evolutionarily conserved protein complex composed of eight subunits [62]. This complex is a crucial integrator of several signaling pathways, acting as a spatiotemporal regulator of membrane trafficking and has been shown to play a role in different processes, including cancer cell invasion, cell migration, ciliogenesis, autophagy, and cytokinesis [63,64]. We recently provided additional insight into the molecular machinery required for melanosome exocytosis by implicating the exocyst in this process (Figure 2). Indeed, we demonstrated that the exocyst complex is essential for melanin secretion and interacts with Rab11b in a mouse melanocyte cell line, upstream of melanin secretion [58]. Importantly, our findings support the model of coupled exo/phagocytosis, since defects in melanocore exocytosis caused by depletion of Sec8 (EXOC4) or Exo70 (EXOC7) exocyst subunits or Rab11b lead to impaired melanin transfer to keratinocytes, causing an accumulation of melanosomes in the melanocyte dendrite tips. This observation implies a defect in melanosome secretion instead of melanin transport, as the phenotype observed is remarkably contrasting to depletion of any of the tripartite complex components, namely MyoVa, Rab27 or Melanophilin, where melanin accumulates in the perinuclear area of melanocytes and its secretion is not affected [44]. Interestingly, the Stow group found the same phenotype upon depletion of Rab11a or Rab11b, as well as Rab17 [40]. Furthermore, melanosome transfer stimulated by Toll-like receptor (TLR)-2 activation is impaired upon Rab11a depletion [65]. Thus, several lines of evidence support the requirement of melanin secretion from melanocytes to achieve melanin transfer and consequently skin pigmentation. Importantly, only the model of coupled exo/phagocytosis involves this requirement.

## 4. Melanin Uptake by Keratinocytes

Despite the crucial role of melanin uptake by keratinocytes for skin pigmentation, the internalization route followed by melanin remains enigmatic. Although several studies suggest that melanin is internalized by keratinocytes through phagocytosis, conclusive evidence is lacking [44,47,50]. Melanosome size varies between 0.5 µm and 2 µm [16]. Therefore, phagocytosis and macropinocytosis are the internalization routes that could allow the uptake of such large cargo [66,67]. Moreover, PAR-2 is one of the few molecular players known to regulate keratinocyte phagocytosis and was shown to promote melanin uptake [34,52,53,68]. Nevertheless, apart from the role in regulating melanin uptake and activation of phagocytosis in keratinocytes, the function of PAR-2 in skin pigmentation remains unclear. Our group demonstrated that keratinocytes internalize melanocores and melanosomes isolated from melanocytes through distinct routes as only the former are PAR-2 dependent (Figure 2) [54]. Since PAR-2 is now well established as a receptor involved in melanin uptake by keratinocytes, our results further support the model of coupled exo/phagocytosis and that melanin is transferred as a melanocore. Nevertheless, it is not known if PAR-2 serves as the receptor for melanocores in keratinocytes.

## 5. Melanin Processing within Keratinocytes

Although melanin disappears upon keratinocyte terminal differentiation in human skin, at least in lower phototypes, it is initially preserved in a process ill-understood [69]. The storage compartment where melanin resides within keratinocytes—the melanokerasome—remains poorly characterized, although previous reports suggest that melanin is stored within lysosomal compartments in keratinocytes [16,33,70]. Autophagy was reported to regulate melanin degradation in primary human keratinocytes [71]. Moreover, autophagy activators reduce melanin levels in human reconstructed skin cultures and skin explants, whereas autophagy inhibitors increase melanin content [71]. Additionally, in kidney tubular epithelial cells, PAR-2 activation was shown to inhibit autophagy via PI3K/Akt/mTOR signaling pathway [72]. Thus, it is possible that a similar inhibition of autophagy occurs after PAR-2-dependent melanin uptake by keratinocytes, allowing melanin to persist within these cells for long periods of time (Figure 2). Considering that PAR-2 regulates melanocore but not melanosome internalization, we postulate that PAR-2 is an essential molecular player also in the determination of melanin fate within keratinocytes. Indeed, we reported that keratinocytes do not have an impaired degradative capacity that could explain why melanin resists within these cells [54]. Moreover, we found that within keratinocytes melanocores are surrounded by vesicles that are positive for early endosome antigen (EEA)1, Rab5, Transferrin receptor, Lysosome-associated membrane protein (LAMP)-2, and CD63, although to a different extent [54]. Therefore, our data suggests that melanokerasomes are either hybrid or transitional early-to-late endosomal organelles. Importantly, melanokerasomes show only moderate acidification and hydrolytic capacity [54], which is suggestive of a storage compartment optimized to retain melanin during the differentiation program of keratinocytes in the skin. This is supported by findings from the Raposo group, showing that melanin clusters are not degradative organelles, as they are devoid of autophagic and highly acidic compartment markers, namely LC3A, Cathepsin V and D, and DAMP [3-(2,4-dinitroanilino)-3’-amino-*N*-methyldipropylamine] [14]. Nevertheless, the presence to some extent of LC3A and Cathepsin V in the upper layers of the epidermis has been previously reported [73].

Melanokerasomes are known to form a supra-nuclear cap or “parasol” over the nuclei of keratinocytes, shielding them from UVR. This localization was shown to be dependent on cytoplasmic dynein and dynactin to aggregate melanokerasomes at the perinuclear region [74]. Also, it was recently shown that the apical distribution of melanin after being transferred to keratinocytes is regulated by the centrosome and centriolar satellites [75]. Importantly, microtubules and actin cytoskeleton networks seem to maintain melanokerasomes in the apical domain of proliferative keratinocytes, protecting these cells from UVR-induced damage. This mechanism can control melanokerasome position during keratinocyte division and impact the distribution of the pigment in the epidermis. Therefore, it is important to also consider the role of keratinocyte polarization and differentiation in what concerns melanokerasome positioning and processing within keratinocytes.

## 6. Conclusions and Future Perspectives

In the past couple of decades, extensive research was carried out to characterize the mode of melanin transfer in the skin. Here, we reviewed the evidence collected, which strongly supports the model of coupled exo/phagocytosis. However, this model could co-exist with others, most likely with the shed vesicles model. To reconcile both models, we propose that the exo/phagocytosis is preferentially used to transfer melanin in homeostasis conditions, whereas under a stress response like UVR exposure-induced tanning, melanocytes can favor other mechanisms of transfer in an attempt to rapidly feed keratinocytes with more melanin, to increase skin pigmentation. Moreover, during the tanning process, melanin could be transferred from melanocytes to keratinocytes but also between keratinocytes in the upper layers of the epidermis as a first response mechanism. Since melanin is a known reactive oxygen species (ROS) scavenger and UVR is known to increase ROS, it is tempting to speculate that keratinocytes exchange melanin to protect more affected regions, while melanocytes upregulate melanin synthesis in response to the oxidative stress. Future studies should continue to test each model in different situations, such as basal conditions, oxidative stress, or upon UVR stimulation. Furthermore, more physiological settings, such as reconstructed human skin epidermis models, ought to be promoted, as the skin architecture is key for its functions. A major flaw in the field is the variety of *in vitro* models used, from melanoma cell lines to animal cell lines, including mouse, chicken, guinea pig, and invertebrates that have very distinct pigmentary systems from humans. For example, mice do not contain epidermal melanocytes and, therefore, melanin transfer only occurs in the hair follicles. Therefore, the variability between models and the intrinsic differences between the pigmentary systems of each model and animal used need to be carefully considered. Although remarkable progress has been made in recent years, there is a need for more physiologically accurate approaches to be developed to unequivocally address this enigmatic topic. This is crucial to develop new and targeted approaches to modulate skin pigmentation in hypo/hyperpigmentation conditions, which can also have important cosmetic applications.
